# Hospital and Institutional News

**Published:** 1916-07-15

**Authors:** 


### HOSPITAL AND INSTITUTIONAL NEWS.

### VISIT OF THEIR MAJESTIES TO THE SOUTH AFRICAN HOSPITAL.

THE King and Queen visited the South African Hospital at Richmond Park on Saturday, July 8. The visit was an informal one, and only short notice of it was given. The hospital has only recently been put into commission, and many of the wards are not yet occupied; it is only fourteen weeks since the erection of the buildings was begun. The various South Africans scattered through the country are to be concentrated, as opportunity allows, in this hospital. The whole work of cooking, it is stated, is carried out by six women cooks: all the ovens, kettles, milk sterilisers, and soup coppers, are heated either by gas or hot water. From the kitchens the King and Queen went to the operating theatre unit, which is thoroughly up-to-date: they afterwards spoke to every patient personally. The buildings have cost £30,000, and the equipment £8,000.

### OPENING OF THE NEW CHELSEA HOSPITAL BY THE QUEEN.

HER MAJESTY THE QUEEN opened the new buildings of the Chelsea Hospital for Women in Arthur Street, King's Road, on July 11. Special constables lined the street, which was decorated with flags; and women police were, with the same appropriateness, on duty inside the hospital. A guard of honour of pensioners from the Royal Hospital was another happy thought on someone's part. The Queen was received by the Bishop of London, Lady Ilchester (president of the Ladies' committee), Lady Londonderry, Adèle Lady Cadogan, and others. A key of the building was presented by the architect, Mr. Keith D. Young, and a bouquet of orchids, the gift of the Ladies' Committee, by Lady Mary Fox Strangeways. The accommodation of the new building is for eighty patients. In commemoration of Her Majesty's opening of the new hospital, a section has been named the Queen Mary Wards. The plans of the newly-completed buildings were published in THE HOSPITAL last year (July 10, 1915, p. 317).

### QUEEN ALEXANDRA AND THE POLICE.

How widespread is the interest taken by families generally in war work, and the universal desire there is to lend aid to it, was shown at a competition between constables, their wives, and children, held in Regent's Park on the 6th instant. It was arranged by Mrs. Pitt and Mrs. Thorpe, of the London Division of the British Red Cross Society. Sir Edward Henry, K.C.B., Commissioner of the Metropolitan Police, was deeply interested in it, and invited Miss Swift, R.R.C., who gave a prize in the children's section, to judge the exhibits and make the awards. The competition was welcomed throughout the Force, and it is one more proof of Queen Alexandra's unceasing sympathy with the life of the people, their joys, sorrows, and home interests, that Her Majesty should have visited the exhibition and gone carefully through the exhibits. It would be difficult to decide whether the pleasure resulting to Her Majesty or to the members of the Police Force, their wives and children, was the greater. The exhibition appears to have escaped the notice of the Press, though it was one, on its merits, which the public, had they known of it, could not have failed to appreciate and support.

### THE NEED OF DOCTORS FOR THE ARMY.

THERE are several signs of the times which point to very great requirements just at present on the part of the Royal Army Medical Corps for more officers. Within the last fortnight a large number of the enrolled practitioners, both of those of military age, and therefore amenable under the Universal Service Acts, and of those within the new limit (fifty-five years) for voluntary enlistment, have received their month's notice that they may be required for service with the R.A.M.C. It is true that only a proportion of those notified are to be actually called up, but there are other evidences of the need that exists. Thus the Local Government Board, in a communication to Poor-Law authorities, states that the Central Medical War Committee has informed the Board that a considerable additional number of doctors will be required for the Army at an early date, and that it is therefore necessary to consider what further medical men under forty-five years of age can be spared without jeopardising the efficiency of the Poor-Law Service. Quite possibly the Army medical authorities expect a considerable number of temporarily commissioned men to decline to extend their service: there must be a large number who are now approaching the conclusion of two years of strenuous service, and may regard themselves as entitled to retire into civilian life for a time, especially since Parliament has sanctioned compulsion for those who have hitherto done nothing.

### A MINISTER ON THE VOLUNTARY SYSTEM.

THE President of the Board of Education, Mr. Arthur Henderson, M.P., made an interesting speech at one of the local meetings in the county held on behalf of the Northamptonshire Hospital Week Fund. He recalled that twenty-five years ago he had been one of a committee which started the workers' collection on behalf of the Royal Infirmary, Newcastle—a collection, by the way, which has reached immense proportions to-day. Mr. Henderson declared that it was right for workers to be brought into contact with hospital work, for in "the great pressure of workshop life" it was impossible to tell who might not need assistance. Mr. Henderson then referred to the invaluable work performed by the hospitals for the wounded, 1,576 of whom have been received at Northampton Hospital since the war began; and described how, when at Newcastle, after the North Sea battle, he saw every ship and man called out to land the wounded. The strength and organisation of the hospitals depended upon "the silent help of the voluntary workers." The local knowledge and patriotism thus engendered gave to the work that touch of personal sympathy without which it might be in danger of becoming a mechanical routine. There was no fear, Mr. Henderson concluded, that the generosity of the British public would ever fail, or that any living man who had fought for us would have to suffer unrelieved.

### THE UNITED STATES ARMY MEDICAL SERVICE

The reorganisation of the Medical Service of the United States Army is to be effected upon lines defined by the National Defence Bill, which was signed by the President last month. Under the new scheme the proportion of medical officers will be seven to each thousand of the enlisted strength—a proportion which existed at the outbreak of the Spanish War and was again restored in 1908, but has been seriously reduced through gradual increase of the army until the enlisted strength had grown 35 per cent. without the addition of a single medical officer. The period of service required of medical officers before promotion to the rank of captain has been increased from three to five years; no provision is made for any promotion for medical officers above the grade of colonel, except the single position of surgeon-general. Army officers of the Dental Corps are open to promotion to the grade of captain after eight years' service and to that of major after twenty-four years' service, subject to the same examination conditions as obtain in the Medical Corps. The Hospital Corps is in future to be known as the Enlisted Branch of the Medical Department, and several new grades, such as master hospital sergeant and hospital sergeant, are created. Through a reduction in the proportion of privates first class in this department, the pay of 75 per cent. of the enlisted men in the Medical Service will be reduced by about 10 per cent. The official organ of the American Medical Association, from which these facts are taken, expresses the opinion that, as the care of the sick is not particularly attractive work to the ordinary soldier, this reduction will probably increase the difficulty of obtaining recruits of fit character and intelligence for the Medical Service.

### EDINBURGH UNIVERSITY AND WOMEN DOCTORS.

Last week we drew attention to the throwing open of certain general hospitals in London to women students; now a step of a similar nature is being taken at Edinburgh. The University there has long granted degrees in medicine to women, but has not admitted them to its classes in the professional subjects; for instruction in the clinical subjects the women have had to rely on what is known as extra-mural teaching, much of it no doubt excellent, but lacking the prestige of academical status. The influx of women students since the outbreak of war has overcrowded the Women's School of Medicine, and as a result of representations from the women students themselves, the Senate has recommended the University Court to admit them to the University classes provided suitable arrangements can be made. It is stated that the University professors now favour this scheme, and doubtless the falling-off in the numbers of male students has facilitated the reform now being accomplished. The University Court has accepted the recommendation of the Senate, which will therefore shortly be carried into effect.

### THE LIMITATION OF PANEL LISTS.

The Middlesex Insurance Committee has adopted a scheme, which has been approved by the Insurance Commissioners, by which no doctor may have more than 2,000 patients on his panel list. Such a limitation was constantly advocated in The Hospital some years ago when the Insurance Act was newly passed, on the ground that a medical man cannot do justice to his panel patients when the number on his list exceeds that figure or one closely approaching it. Five thousand, and even six and seven thousand, insured persons were actually allowed to be allotted to a single practitioner in those days, and probably are still in many districts. This condition of things is a gross injustice to the insured, and we are very pleased to see that in Middlesex it is not to be tolerated any longer. Another part of the scheme is that the funds available for distribution among the panel doctors in respect of persons who have not selected any doctor shall not benefit anyone whose panel list contains two thousand names or upwards.

### CHARING CROSS HOSPITAL THROWN OPEN TO WOMEN STUDENTS.

Hard on the heels of King's College Hospital, St. Mary's, and St. George's, Charing Cross now announces that the hospital and medical school there are to be thrown open to women students. It is true that the announcement states that this is a temporary measure, due to the scarcity of doctors and the need of skilled assistants in the wards. As recently as 1915 the Council decided against a similar proposal, and it seems to be Professor Halliburton's advocacy that has influenced them to reconsider that decision. It is, of course, only the clinical studies that Charing Cross will provide; in accordance with a scheme which has been working for some years, the instruction in the preliminary sciences will be given at King's College. No limit has been set to the number of students who will be admitted, though it is probable that they will not be allowed to outnumber the male students. Arrangements for the necessary accommodation are now being made, and applications will be received forthwith.

### HOME TRUTHS AT ABERDEEN.

A lady doctor has been arousing the public interest and, we hope, the public conscience in Aberdeen lately in the course of some health lectures delivered under the auspices of the Burgh Insurance Committee. Amongst other home truths, she told the Aberdonians that they have a capacity for drug-swallowing unequalled in the British Isles. They hold the belief that recovery from illness is merely a matter of swallowing large quantities of medicine, and that if only the doctor is clever enough to pick out the right sort for them they can with impunity violate every canon of health and hygiene. The statistics of the Insurance Committee do most undeniably support this indictment. The Aberdeen panel drug account per insured person exceeds that of every other town in the kingdom, and exceeds indeed the maximum allowance sanctioned by the Insurance Commissioners. Nor is this the only rebuke offered to Aberdeen; it is also said that parents allow their children up far too late in that city and that the health of the latter suffers in consequence of the neglect of their parents to enforce proper hours for going to bed. Altogether Aberdeen is being startled, to judge by the comments of the local Press; we trust that good results will follow.

### THE CHAPLAIN IS WORTHY OF HIS SALARY.

AFTER a keen debate the Sheffield City Council referred back a proposal to pay £75 to the Church of England chaplain, £35 to the Nonconformist, and £20 to the Roman Catholic clergy for their duties as chaplains at the infectious diseases hospital. It was a Roman Catholic councillor who reminded the meeting that the clergy had to live like other men, and that it was "pious cant" to suggest that men would desire to give valuable services for nothing. No professional people ever did. Equality of payment was advocated by another speaker, but while it is certainly true that chaplains should be duly paid like other people, we conclude that the different fees are based upon the respective amount of work demanded of the different denominations.

### A ROMAN CATHOLIC SANATORIUM.

SECTARIAN institutions are fewer than they once were, but the first annual report has been issued of St. Joseph's Children's Sanatorium, at Freshfield, near Liverpool. Here boys from three to eight years old, and girls from three to fourteen, are admitted and placed in charge of the Poor Servants of the Mother of God. The report states that thirty-seven children, all of whom were in the earlier stages of tuberculosis, have been treated, and that the Local Government Board has approved plans for extension, which depends upon the support given. The report, which has been issued by Mgr. Canon Pinnington, deals with the work undertaken since the sanatorium was opened on March 27, 1915.

### SUICIDE OF A SWANSEA HOSPITAL PATIENT.

A RATHER curious case has been investigated by the Swansea Deputy-Coroner. A nurse, giving evidence, said the patient underwent an operation, and, although in great pain, seemed fairly comfortable afterwards. She had occasion to use a razor for the dressings in certain cases one Friday afternoon, and she left it on the dressings-trolley in the middle of the ward, about three yards from the patient in question. She left the ward to serve dinner, and upon returning found that the patient had jumped out of bed, seized the razor, and inflicted a gash in his throat. Dr. Londen said the cut in the throat was superficial and not sufficient to cause death, which was due to collapse and shock following a severe physical effort when the patient was in a weak state of health. The jury's verdict, however, was "Suicide while temporarily insane."

### INFANT HYGIENE IN WALES.

As Sir William Osler pointed out very forcibly in a number of addresses which he gave at various centres in the Principality a few days ago, Wales is a black spot in regard to its infant mortality, which is abnormally high. Many of the Welsh health authorities are woefully indifferent to their responsibilities, and some additional spur seems to be needed to awaken them to action. One is glad to note that the Penybont Rural District Council at Bridgend last week decided to appoint two health visitors at a salary of £100 per annum each. There is urgent need of many more of these useful officers in Wales, especially in the southern half of the Principality. In view of the high infantile death-rate which prevails in the borough, the Merthyr Health Committee is being urged by the Local Government Board to increase its staff of visitors. Merthyr should certainly take some steps to wipe out the scandal of its infant death-rate.

### THE SCOTTISH WOMEN'S ORGANISATION.

THE Scottish Women's Hospitals for Foreign Service, it appears, are sending further help for Serbia beyond that mentioned in the last issue of THE HOSPITAL. At the request of the Serbian Government and the British Army medical authorities the organisation is providing three new hospital units for attachment to the Serbian Army. Two of these units, 200 beds each, will be in charge of Dr. Elsie Inglis and Dr. Bennett, and the third—a motor-ambulance flying corps—will be superintended by Mrs. Harley. At home another hospital will be opened early next month by the Scottish branch of the Red Cross—Ralston Hospital, a permanent home in lovely surroundings for sailors and soldiers who have been totally paralysed through injuries received in action.

### INTOXICANTS FOR MILITARY PATIENTS.

AN extraordinary case was heard at the Ormskirk (Lancs) Police Court last week, when a widow of fifty-six, described as a lady of means, was sent to gaol for three months without the option of a fine for giving intoxicating liquor to wounded soldiers. The defendant lived in close proximity to a large military hospital at Maghull, near Ormskirk, and was in the habit of inviting wounded soldiers to her house to tea, and then giving them intoxicants. A sergeant said he stayed at the house eight days, and another soldier gave evidence that he remained overnight until 10.30 the next morning, both being supplied with drink. The woman, who denied the whole story, pleaded hard for a fine.

### RED CROSS COUNTY HISTORIES.

THE Warwickshire Branch of the Red Cross Society has done well to issue a history of its work since its foundation in 1909. The author is Brig.-General M. Quayle Jones, who was himself County

Director until December 1914, when he relinquished the post for active service. As a matter of fact, before 1909 there was a branch already in the county, but it was then that the Territorial Force Association was asked to hand the raising of V.A.D.s over to the local branch of the British Red Cross Society, under the supervision of a County Director. General Quayle Jones does well to record the early beginnings of the work in Warwickshire. He was succeeded by Mr. W. H. Wykeham-Musgrave, who retired, also for military work, in June 1915, when Mr. E. H. Little was appointed. There are now twenty-three hospitals controlled by the branch, and since the date of the declaration of war sixteen new detachments have been created, making a grand total of fifty-two for the whole county. About three-quarters of these have been mobilised. The total cost of maintenance has been £18,701. The record of the work thus outlined modestly calls itself a report, but General Quayle Jones has really sketched a county history, and he and his publishers have been generous with illustrations, which are a feature of a carefully prepared report.

### MANCHESTER HOSPITALS AND THE BATTLE OF THE SOMME.

MANCHESTER, stated to be now the biggest war hospital centre in the kingdom, experienced the busiest time of all the war during the early part of last week. The large arrivals of men wounded in the new offensive caused a great deal of activity in the base hospitals and the fifty auxiliary Red Cross hospitals in and around the city, which have a grand total of 15,000 beds. The Whitworth Street Hospital was emptied and the patients transferred elsewhere, and the Worsley Hall and High Street Hospitals were likewise evacuated and made ready for wounded officers. The finely equipped transport department of the local Red Cross branch transported in the three days over 3,000 wounded from ambulance trains met at Manchester, Chester, and Warrington; seventy ambulances and 300 motor-cars were employed in this work.

### UNUSUAL ACTION AGAINST A NURSING HOME.

AN unusual case is before the Chancery Division in Ireland, in which an injunction is sought to restrain a tenant from using certain premises as a nursing home. It appears that the property belonged to a Roman Catholic priest, who let a house to a nurse on covenant to use it strictly as a dwelling-house and not for any business. The lady, however, established and carried on a nursing home there; it was only after the owner's death that objection was taken by his trustees, also priests. The ground of objection was the usual one in such cases, that by using the premises for business purposes the value of neighbouring property is deteriorated. For the defence it was urged that the establishment of the home had not only not deteriorated the property, but that it might even have the opposite effect. It was also stated that other houses in the same street had been converted to business purposes without let or hindrance from the lessors.

### THE SURBITON RED CROSS HOSPITAL SECRETARYSHIP.

THE resignation of Mr. A. G. Gumpert from the position of honorary secretary to the Kingston, Surbiton and District Red Cross Hospital has led to the appointment of Mr. W. Acheson Gray, the hospital's honorary treasurer, who is to hold both positions. Mr. Gumpert had acted as honorary secretary since the hospital was opened a year ago next month, and has been formally thanked for his services. It is not generally a good plan for the same person to act as secretary and treasurer, whether in an honorary capacity or not, and in so far as it was found necessary for the two posts to have been held by two persons for the first ten months, a change now seems to deserve an explanation.

### THE GENEROSITY OF LOTHIAN MINERS.

AT a meeting of the Edinburgh Royal Infirmary Board of Management it was reported that the Mid and East Lothian miners, whose ordinary subscriptions are at the rate of a penny per fortnight per man, have decided to double their contributions to the institution during the six months ending December next. The receipt was announced of a sum of £1,000, being a first payment on account of the proceeds of the "Infirmary Badge Day" held on May 27.

### CLEARING OFF DEBT AT MANSFIELD.

THE board of management of the Mansfield Hospital, to which was recently added the King Edward Memorial Wing at a cost of £15,000, and a Nurses' Home costing £4,000, have issued an appeal to the colliery and royalty owners in the district to assist in clearing off the debts on the hospital and to increase their annual subscriptions. A generous response was at once forthcoming from the Bolsover and the Sherwood Colliery Companies, which, in addition to contributing £1,000 each towards the debt on the capital account of the extensions, have doubled their yearly subscriptions. These subscriptions are now £200 and £100 respectively.

### THE EXTENSIONS AT WIGAN.

NOTWITHSTANDING the difficulties created by the war, the new out-patient department at the Royal Albert Edward Infirmary, Wigan, is nearing completion. The need for the new department has been insisted on, we believe, for ten years, and it really forms part of a scheme which will include an up-to-date operating theatre. On the out-patient department a sum of £12,000 is being spent, of which one-sixth remains to be subscribed, so that there is a reasonable chance of it being opened free of debt. When this has been done, the workers, who subscribed £8,500 last year, will be asked to club together to find the £1,500 required for the new theatre. They have not been specially approached in reference to the larger sum, but as a thank-offering for the presentation by others of the new out-patient department, which will benefit them, an effort to pay for a new theatre would be timely and of great value.

### SURGICAL TUBERCULOSIS AT NEWPORT.

INHABITANTS of the locality have protested by deputation to the Newport Health Committee and to the Corporation against the proposal of the Welsh National Memorial Association to utilise the freehold detached residence known as Cardigan House, Clytha Park, as a hospital for the treatment of surgical tuberculosis. The ground of their objection is that such an institution ought not to be set up in a purely residential district of Newport, and their contention, though it is obviously dictated by selfish interests, seems to have weighed with the Health Committee, for they were equally divided upon it. Cardigan House was recently given to the Memorial Association for the purpose named by Sir Garrod Thomas. It is "up to" the local medical profession to educate both the Clytha Park residents and the Newport Health Committee.

### PUBLIC LIBRARIES AND TUBERCULOSIS INFECTION.

AT Portsmouth the Sanatorium Sub-Committee of the local Insurance Committee has been impressed with the possible risk of the spread of tuberculosis through books issued at the public library; and has been in correspondence with the Libraries Committee of the Town Council on the subject. The former body has invited the latter to consider whether it is possible to prevent the issue of books from the lending department to persons suffering from tuberculosis in an infectious stage. The difficulty appears to be that it is not legal for the medical officer of health to supply the Libraries Committee with a list of persons notified as tuberculous; it is stated that in this respect tuberculosis stands on a different footing from other notifiable diseases, and that only an alteration in the law will permit the Portsmouth authorities to carry out their scheme. Alternative suggestions made were that all the books should be disinfected, and that a special department should be created at the library for the use of tuberculous patients. Neither of these is very practicable, so the matter for the present has been referred to the Sanatorium Sub-Committee for consideration and report.

### THE PAY OF POOR-LAW DOCTORS SERVING AT THE FRONT.

EXCEPTION was taken at a meeting of the Derby Board of Guardians to the amount of the grant made to certain officers of the Board, including the medical officer (Dr. A. D. Hunt), now serving with the Colours. The sum granted to Dr. Hunt is £100. It was urged that while the patriotism of the officers was worthy of recognition, the Guardians should not waste money in grants when they were economising in every other direction, and that the case would be justly met if the total income of the officer, including his Army pay, were made equivalent to his former salary. A motion in these terms was proposed, but it was defeated by an overwhelming majority. One of the members said Dr. Hunt saved the Board quite £100 by undertaking extra duty before he went on medical service.

### MARGARINE VERSUS BUTTER.

MARGARINE has now been used instead of butter at the Belfast Poor-Law Infirmary for three months. The result has been most satisfactory: £345 has been saved, and apart from a protest at the beginning of the trial there has been no complaint. When the substitution was first made, the medical officers were authorised to order butter for any patient for whom they considered it desirable. This power has not been exercised by any of the medical officers. Dr. John M'Liesh reported on the matter on behalf of the medical staff, and said that up to the present they had observed no injurious effects from the use of margarine upon the patients in the infirmary.

### GUARDIANS' SUBSCRIPTIONS TO HOSPITALS.

AFTER considerable discussion the Borsmore Guardians have decided to double their usual subscription of ten guineas to the East Suffolk and Ipswich Hospital. This decision was arrived at in dealing with a direct request from the institution, and the only point on which an amendment was moved centred on the question whether an increase should be a special donation, a second subscription, or a new subscription at a higher rate. It is noteworthy that one speaker admitted that the poor of whom those present were the Guardians benefited greatly by the institution, while the note of opposition from those who thought the ratepayers were doing all they could or that hospitals should be rate-supported did not deal with the constructive policy which is open to local authorities under the Public Health Acts. It has always seemed to us that the issue for Guardians is a simple one, to be decided by the number of the poor from their district who are actually treated at the institution which asks them for a subscription. If their poor are cared for at the hospital, even from the point of view of those who advocate a municipal system, the inadequacy of the public hospitals is proven, and consequently the support of those institutions which are actually doing the work becomes the most serious of duties. We are glad to note that it is being increasingly recognised.

### THE JULY "GAZETTES."

THE 1st Eastern General Hospital, Cambridge, has entered on a new development with the departure of its registrar and other members of the staff to form the nucleus of a new base hospital in the East. The recent issue of the hospital's *Gazette* in which this is recorded refers also to the attempts at ward gardening which have been carried out, chiefly, it seems, at the personal instigation of a sister who has now left. The *Gazette*, which confines itself almost wholly to the lighter side of war hospital journalism, is in striking contrast to the fat magazine on thick white paper issued by the Craigleith Hospital. Here the aim evidently is to interest by information, and the series of articles on regimental histories, and the sketch of life "In a French War Hospital," are worth reading. Few hospital *Gazettes* are better produced than this of the Craigleith Hospital, which is well worth examination for this point of view alone. The July number of the 3rd London and the "Southern Cross" have no new features of special interest.

### THE LATEST WAR HOSPITAL GAZETTE.

With July is issued the first number of the *Huddersfield War Hospital Magazine*, edited by Major E. G. Coward, R.A.M.C. As there are nine affiliated institutions, and the hope is expressed that "many incidents from the Front which would otherwise have lain dormant" may be published, there should be plenty of good material for future issues. The green cover bears a design of oak-leaves and acorns, for which Miss Prescott is responsible. Inwoven with them is the town crest, as a memorial of the local generosity which presented the hospital to the War Office, and the R.A.M.C. badge and motto, the latter of which, as we have already pointed out, is a good motto for editors. Lieut.-Colonel W. L. W. Marshall, the officer commanding the hospital, has offered a monthly prize of 10s. for the best article or poem, and a similar prize for the best "work of art reproduced in its pages." The awards will appear next month. Perhaps the most remarkable contribution is the first instalment of a series of extracts concerning the retreat from Mons to Ypres, taken from the diary of a former patient, Rifleman A. Liney, K.R.R.C. The price of the magazine is twopence.

### STREET COLLECTIONS FOR HOSPITALS.

There was more than a grain of truth in the remark made by Sir T. Vesey Strong last week to the effect that the British public is beginning to feel it is being pressed a little too far in the matter of street collections. This opinion, already voiced by The Hospital, was expressed at a meeting of the Board of Delegates of the Hospital Saturday Fund in response to a suggestion made by the secretary of the Bethnal Green Committee that, in view of the closing down of certain railway stations, official permission should be applied for to enable the Fund's collectors to go into the street. When the Fund's street collections were discontinued in 1897 the total from all sources was about £21,000, while last year the income was over £46,000, and it is expected that this year it will exceed £60,000. Under all the circumstances, therefore, it would seem to be a wise policy to "let well alone."

### HERB GARDENS IN PUBLIC PARKS.

Some practical interest really seems now to have been aroused in the development of medicinal herbs in England. This takes the two forms of collecting, under proper supervision to avoid waste, wild flowers from which remedies are obtainable, and also of growing valuable herbs in private and public gardens. The herb garden which belonged to the late Sir Spencer Wells still flourishes in Golder's Green Park, and is said to contain every herb mentioned by Shakespeare. Surely, we may add, a herb garden would be a valuable and popular feature of every public park. Dutch gardens, Japanese gardens, and rock gardens are already part of the decorative features of many of them, and a herb garden can be equally decorative; and the scent! Meantime, till this educational undertaking be adopted, it may be helpful to note the chief agencies already at work moulding public opinion, and the principal sources of information obtainable. The Royal Horticultural Society has published a pamphlet on medical herbs, which is sold by W. Wesley, 28 Essex Street, W.C., price 6d. It gives useful hints on collecting and drying. Then there is the monthly circular of the Herbgrowing Association, whose offices are 7 Queen Anne Chambers, Westminster, which gives orders to its members and accepts supplies from them for the wholesale dealers. It charges a commission on the price obtained. Then, of course, there is leaflet No. 288 issued by the Board of Agriculture and Fisheries. These are helpful agencies at the present time; but the parks, which have taught Londoners so much about the habits of birds and the delights of formal gardens, might well revive the old-fashioned herb gardens as another charming and educational feature.

### THIS WEEK'S DRUG MARKET.

The quieter tone which has been noticeable in the drug market during the past few weeks is still in evidence. Very few drugs are attracting special attention, and such price fluctuations as there have been have not been violent. Importers of quicksilver having raised their price, quotations for calomel, corrosive sublimate, and other salts of mercury have been advanced. There is no change in the position of bromides, and there is perhaps a firmer tone in this market. Salicylates are again lower in price, and the general impression is that the lowest figures have not yet been touched; it would appear to be unwise to purchase sodium salicylate and salicylic acid beyond immediate requirements. Acetanilide and acetylsalicylic acid both have a slightly lower tendency in price. Supplies of phenacetin are scarce, and the price continues to advance. Salol is rather cheaper. A large business continues to be done in camphor, and prices have an upward tendency. Opium is unchanged; the values of morphine and codeïne are maintained. Quinine is unchanged.

### TO OUR READERS.

Contributions are specially invited from any of our readers to these columns. **They should deal with topical subjects and news. They must be authenticated for the information of the Editor only. The minimum payment if published is 5s. There is no hard-and-fast rule as to space, but** notes of about twenty lines in length are preferred.

